# Applying an Ethical Framework to Herbal Medicine

**DOI:** 10.1155/2018/1903629

**Published:** 2018-09-19

**Authors:** Kate Chatfield, Bahare Salehi, Javad Sharifi-Rad, Leila Afshar

**Affiliations:** ^1^Centre for Professional Ethics, University of Central Lancashire, Preston, UK; ^2^Medical Ethics and Law Research Center, Shahid Beheshti University of Medical Sciences, Tehran, Iran; ^3^Student Research Committee, Shahid Beheshti University of Medical Sciences, Tehran, Iran; ^4^Phytochemistry Research Center, Shahid Beheshti University of Medical Sciences, Tehran, Iran; ^5^Department of Chemistry, Richardson College for the Environmental Science Complex, The University of Winnipeg, Winnipeg, MB, Canada; ^6^Department of Medical Ethics, Shahid Beheshti University of Medical Sciences, Tehran, Iran

## Abstract

Herbal medicines make a vital contribution to healthcare globally, but from production through to practice, there are ethical challenges that require attention. Ethical challenges are often analysed through application of an ethical framework because this can facilitate a consistent and structured approach. In healthcare, the most commonly used framework over recent decades has been that of the four principles: beneficence, nonmaleficence, autonomy, and justice. However, for various reasons that are explained, this approach to ethical analysis is not the most fitting for the global phenomenon of herbal medicine. In this paper, a relatively new moral framework that is based upon the globally accepted values of care, respect, honesty, and fairness is explored in relation to herbal medicine for the first time. Through application of this framework, the ethical challenges and actions needed to address them become clear, thus resulting in practical recommendations for enhancing ethical standards in herbal medicine.

## 1. Introduction

Plant-based herbal medications have been valued by cultures around the world since ancient times, and globally an estimated 70,000 species of plants are used for medicinal purposes [1]. However, over the last thirty years or so, a number of potential risks associated with the use of herbal medications have received growing attention. In particular, there are concerns that only a tiny fraction of used species have been subjected to rigorous testing under controlled conditions, and clinical studies of herbal medicines vary greatly in quality and usefulness [2]. The aim of this paper is to show how the application of an appropriate ethical framework can reveal both the ethical challenges and the actions needed for enhanced ethical standards in the production and usage of herbal medications.

Herbal medicine, also known as botanical medicine or phytotherapy, involves the use of a plant's seeds, berries, roots, leaves, bark, or flowers for medicinal purposes, and the way in which plant-based medicines are used varies greatly around the world. Herbal medicine can be considered as either a traditional or a complementary form of treatment, depending upon where and how it is used. The World Health Organization (WHO) distinguishes between healthcare interventions that are used in a complementary manner, away from their geographical origins, and those that are rooted in local tradition and culture. According to the WHO, any form of healthcare that is indigenous to a particular region can be classified as ‘traditional medicine' within that locality, but outside that region it may be considered as ‘complementary medicine' [3]. In high income countries, most nonconventional medicines are used in a complementary manner and people who can afford to pay for additional services choose from a broad range of privately delivered treatments. In low and middle income countries, many forms of traditional medicine are region-specific, and the types that are available are largely dependent upon the accessible flora, fauna, and other local resources, as well as the local traditions. When used in a traditional manner, the types of herbs depend upon the plant species that are local to that region. Hence, the plants used in traditional herbal medicine in China will be mostly different from those used in traditional treatments in African countries [4]. Herbal medications contribute significantly to most indigenous forms of medicine, termed ethnomedicines, and their use is often intertwined with local customs and beliefs about the workings of the body and the nature of health and disease [5]. Some types of ethnomedicine, such as Traditional Chinese Medicine or Ayurvedic medicine, incorporate a range of therapeutic interventions which are used alongside herbal treatments to form a holistic package of care. However, in the West, herbal medicine is more commonly viewed as a discrete therapeutic approach, even though it is often used at the same time as other treatments (such as conventional medications). When used in this complementary manner, the herbs may be sourced locally or they might be imported from across the globe. For instance, herbal treatments from Africa or China could be used alongside conventional or other treatments in a European country.

The WHO suggests that usage of traditional medicines may be as high as eighty per cent of the population in some parts of Asia and Africa where many people rely upon some form of traditional medicine for their primary healthcare [6]. More recently, this high figure has been called into question [7], but there is little doubt that the global market for herbal medicine is substantial. In 2016 this was valued at 71.19 billion US dollars and this figure is predicted to rise in the near future [8]. The increasing market has been attributed to a growing preference for ‘natural' medicines such as Ayurveda, Unani, and Traditional Chinese Medicine which are largely comprised of plant-based medicines.

Reported reasons for the increasing the popularity of herbal medicines as a complementary treatment amongst Western consumers have included health promotion [9], avoidance of side effects from conventional treatments [10], and lack of effective conventional care [11]. Aside from their desirability as a traditional or complementary treatment, herbal medicines also contribute directly to the development of conventional medications with as many as one-third to one-half of pharmaceutical drugs being derived from plants [12]. For example, the drug* Vinblastine* is derived from Periwinkle, a plant that is commonly found in Europe and North Africa. This drug is primarily used as a chemical agent in many types of cancer. Additionally, with grand challenges like drug resistance, depletion of fossil fuels, and the impact of ecological degradation threatening the wide scale production of conventional drugs, the need to investigate the full potential of plant-based alternatives has become a growing imperative [13–32]. The vital contribution of plant-based medicines to our pharmacopoeia for both current and future generations is undeniable. Still, given that most herbal medicines are underresearched in terms of efficacy, safety, and cost effectiveness, this inevitably generates ethical concerns.

## 2. The Primary Ethical Challenges for Herbal Medicine

A significant number of people hold the mistaken assumption that herbal products are safe because they are natural [33, 34], but herbal medicines have pharmacological effects, just like synthetic pharmaceuticals. Many plants are potent or toxic and there is typically far less safety data available for herbal products than would be required for conventional medications [35]. Some herbal medicines have been associated with adverse drug reactions including overdose and toxicity [36, 37], drug/herb interactions [34, 38], allergic reactions [39], and contamination with other products [40, 41]. Additionally they have been known to interfere with results from laboratory tests [39]. In most regions of the world, consumers have access to unlicensed herbal medicines [42], which may expose them to any of these dangers without the oversight of an experienced practitioner.

Unlike pharmaceutical drugs, most herbal medicines are not taken through the phases of development before release onto the open market. In fact, most herbal medicines have been in use for substantial periods of time, perhaps even hundreds or thousands of years, before they are subjected to clinical studies, if at all. It may seem reasonable to assume that the extensive use of herbal medicines over long periods of historical duration should reveal all implications for their safety. Information about who might be at risk, how much to take, and potential side effects can be gleaned from years of real world experience and guidance developed accordingly. This type of information evolves as it is passed down through generations and is vital to locally based, traditional forms of medicine. However, when products are removed from their cultural and traditional roots and applied in completely different environments, under different conditions, and in different formats, the consequences of such use are unpredictable [3]. Even those herbal medications that are relatively well-studied may not have been tested with certain people, such as paediatrics, pregnant or lactating women, elderly people, or those with multiple morbidities [43].

Because most herbal medications are underresearched, their potential for harm is often unknown [44] and this has a direct impact upon the informed consent process. When physicians are unable to provide evidence about efficacy or the potential for side effects, patients cannot be fully informed about risks and benefits [45, 46]. Perhaps even more alarming is the allegation that information about herbal medications can be dangerously misleading [38, 47]. In addition to the potential for harm from the medications, there are also concerns about the providers of herbal medicine because, in many environments, they are not medically trained [44], and neither are they well regulated [48].

Aside from the impacts upon people, the production and delivery of herbal medicines has impacts upon local environments and communities. For instance, the growing demand for standardised herbal products is putting pressure on selected high demand species [49] and some plants are in danger of extinction as a result of demand [50]. A demand-oriented market and poor quality checks have motivated certain producers to go for mass production without taking into account the consequences of plant cultivation. Furthermore, many medicinal plants are collected from the wild in an uncontrolled manner because cultivated plants are often considered inferior [51]. Unsustainable harvesting threatens the survival of not only medicinal plant species, but also the people that depend upon them [52]. Along with the growth in global markets, there has been an increase in biopiracy, whereby traditional herbal medicines have been patented without consent or compensation to their holders [12].

It would be both unrealistic and unethical to restrict use of plant-based medicines to those that have been tested robustly as this would deny access to medicine for entire communities, particularly in resource-poor areas. In higher-income regions, where herbal medications are used in a complementary manner, restriction would compromise respect for individual preferences. Nevertheless, given the aforementioned ethical challenges for the provision of herbal medicine, it is vital that measures that are respectful of individual needs and preferences are put in place while avoiding harm to patients, the environment, and local communities.

## 3. An Ethical Framework for the Provision of Herbal Medicine

Ethical evaluation is often conducted through the application of an ethical framework because frameworks can facilitate a consistent and structured approach. An extremely large number of ethical frameworks have been developed. Some are aligned with particular ethical theories, such as the consequentialist framework, the duty framework, and the virtue framework [53], while others have been developed for use within certain professions.

Most people who work in healthcare will be familiar with a framework that is underpinned by the four ethical principles of beneficence, nonmaleficence, autonomy, and justice. Together they stipulate that healthcare professionals must aim to do good, avoid doing harm, respect the right of individuals to make decisions for themselves, and ensure that people are treated fairly [54]. This approach, known as* principlism*, has been prominent in biomedical ethics since first introduced by American scholars, Beauchamp and Childress, almost 40 years ago. Undoubtedly, principlism has proven worth, as evidenced by how it has been adopted across healthcare professions, and beyond, to guide professional practice and research. However, principlism has received a fair amount of criticism, being perceived by many as being ‘explicitly American in nature' [55] and hence not readily applicable across cultures. For instance, the principle of autonomy, which is interpreted by Beauchamp and Childress as the right of individuals (who have the mental and physical capacity) to make decisions for themselves, is not recognised in all cultures globally; in some cultures, decisions about healthcare are reached by consensus, rather than by the individual [56].

**Figure 1:**
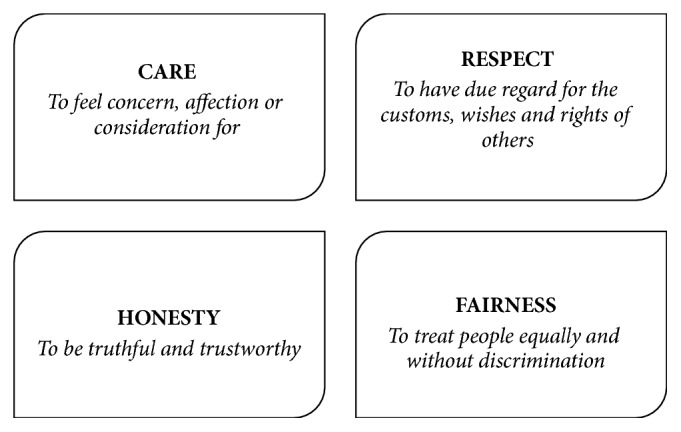


Given that the production and use of herbal medicine is a global phenomenon, we propose application of an ethical framework that has worldwide applicability. Recently, such a framework has emerged from the work of a global consortium of researchers, academics, research participants, policy makers, and research ethics committee leads together with representatives from funding bodies and the pharmaceutical industry. Their findings suggest the use of a framework that is based upon values rather than principles, namely, the values of* care, respect, honesty,* and* fairness *as shown in Figure 1 [57].

Principlism has been accused of taking a rather formulaic approach to ethics that focusses upon the application of processes rather than upon ‘being ethical' because it does not take any account of the virtue and intentions of the person acting [58]. This is not the case with a values approach. Values describe what people believe to be important and, significantly, they are recognised as a driving force in decision-making. Hence, the values one holds will influence the kinds of decisions that one makes. Values can vary across cultures but these particular values have been found to have cross-cultural agreement and understanding. The values framework was originally developed for application in research collaborations and has been used to develop two codes of conduct for research [57, 59] but the four values can equally provide a foundation for analysis of ethical concerns across a broad range of disciplines and scenarios, including the practice of herbal medicine. At any point in time, those who work in herbal medicine, in whatever capacity, can reflect upon whether they are acting with care, respect, honesty, and fairness. The idea being that, when people hold true to these values, ethical standards are upheld. Being a relatively new moral framework, the full potential of this approach is yet to be explored and in this paper it is considered for herbal medicine for the very first time. In the following section, the values are further described with examples of how they might be enacted in herbal medicine to ensure high ethical standards.

## 4. Application of the Four Values to Herbal Medicine

### 4.1. Care

Care in herbal medicine is demonstrated through concern for the welfare of those who use the medications as well as the communities and environments from which they are sourced. For practitioners, care requires knowledge and expertise; practitioners must make judgements about when particular medications are indicated, are appropriate, and are safe. When practitioners are working with incomplete or inaccurate information, this can lead to harm. Hence, a crucial component of care is a commitment to life-long learning; practitioners should stay abreast of new developments and safety information. Additionally, care is shown when practitioners work only within their bounds of competence and use products from trusted suppliers to ensure quality and ethical sourcing methods. A further example of care might be seen in the way in which practitioners communicate with their patients. For instance, the provision of written information to an illiterate person exemplifies a distinct lack of care. For producers of herbal medicine, care entails the preservation and conservation of local environments and avoiding harm to local communities. Of course, there are challenges when acting with care; it may require more time, more effort, better resources, and easy access to the appropriate information.

### 4.2. Respect

Respect requires acceptance that people's preferences, customs, and cultures may be different from one's own. In healthcare, it can mean that a provider or practitioner accepts a way of approaching treatment or a decision that they would not select for themselves.

In cultures where there is traditional usage of herbal medicines, certain treatments may hold cultural or spiritual significance for entire communities that are not obvious to outsiders [60]. There might be greater trust in medications that have centuries of historical usage [61]. Additionally, decisions about healthcare may be taken by particular members of a community rather than by individuals, such that respect for different customs and decision-making processes is necessary [61].

For individuals who use herbal medicine in a complementary manner, healthcare providers should respect that individuals can ‘pick and choose' a variety of treatments for different ailments. This may lead to challenges, especially when there are differing opinions about what will be most helpful to the patient.

### 4.3. Honesty

In all cultures and nations, ‘do not lie' is a basic prerequisite for ethical human interaction. However, the value of honesty has a broader scope in the context of healthcare. Deliberately lying is an obvious wrongdoing, but it is equally unacceptable to omit important information that is necessary for informed consent. Hence, herbal practitioners should be honest and open about the type of evidence that is available for their treatments in terms of efficacy and the potential for adverse effects. Gaps in knowledge need to be revealed. Equally, honesty is necessary in promotional activities. It would be unethical to mislead patients or promise cure in circumstances where there is no certainty of benefit. For herbal practitioners, honesty requires clarity about when evidence stems purely from traditional usage and when traditional knowledge has been tested through scientific investigation.

### 4.4. Fairness

Fairness can have a number of interpretations but the most relevant concepts for ethics in herbal medicine are* fairness in distribution*,* corrective fairness*, and* fairness in exchange*. Fairness in distribution concerns the availability of treatments. It implies that the same quality of treatment should be available to all, regardless of economic or social status, or any prejudicial factors. Of course, we know that this is not currently actualised in healthcare. The very reason why so many people rely upon herbal treatments in resource-poor settings is because they offer the only affordable and accessible choice [62]. In environments where people have the ability to choose herbal medicine as a complementary treatment, high quality treatment may be priced beyond reach of many. While practitioners are not in a position to address the grand challenges that underpin inequity in availability, they can enact fairness at a local level when they do their best to treat people equally and without discrimination. Concerning the other two types of fairness: corrective fairness is about how to right a wrong and includes considerations such as liability and accountability, and fairness in exchange might include the fair charging for services.

## 5. Recommendations for Enhanced Ethical Practice in Herbal Medicine

Even from this very brief consideration of the four values in relation to herbal medicine, specific challenges for the realisation of high ethical standards are revealed. Some of these, such as the unequal distribution of quality and availability around the globe, are beyond the influence of most. However, there are smaller scale, pragmatic solutions that lie within the realms of possibility for various stakeholders.

Practitioners can exemplify the four values in their practice. When they reflect upon whether they are acting with care, respect, honesty, and fairness, shortcomings in ethical practice may be revealed. For instance, they may conclude that more effort is required to keep their knowledge up to date and to contribute to international reporting schemes for adverse effects such as that of the WHO Programme for International Drug Monitoring.* VigiBase*, the WHO global database of individual case safety reports, relies upon the reporting of adverse effects by individuals and practitioners to monitor safety of all drugs, including herbal medications [63].

For educators, inclusion of a values approach in their teaching may help to reveal areas where they can contribute to the improvement of ethical standards. Knowledge and understanding of herbal medications form only one component of what is necessary for ethical care; there are many other considerations for ethical practice. Furthermore, because the nature of evidence for most herbal medicines is of such a different quality to that of drugs in conventional medicine, educators need to address what happens when there are evidence gaps or variations in reliability. Aside from the education of herbal practitioners, universities with medical, nursing, and pharmaceutical programmes can help to improve ethical standards through development of curriculum on the use and safety of natural therapeutic products. This would enable them to discuss the use of herbal medications with their patients in an informed manner.

Manufacturers can also implement the four values, taking care to produce products that are of reliable quality and free from contamination. Honesty requires the correct labelling of products and reliable advertising. Fairness would ensure reasonable pricing as well as reasonable recompense for the community where the herbs are sourced. Both respect and care are also needed for ethical sourcing of herbs to ensure that harm is not caused during the growing, harvesting, and manufacturing of herbal products.

Lastly, it is clear from this analysis that ethical standards are compromised greatly by deficiencies in research into herbal medications. When information is limited, practitioners can be working in the dark and patients may have insufficient information for informed consent. For fully informed consent, patients must understand the implications of using herbal medications [64], but in situations where there is little or no evidence for efficacy or safety, it is impossible for medics to provide accurate and reliable guidance [65].

Herbal medicine research can provide much needed information about effectiveness and safety that can be transmitted to the clinical environment and be used to inform patients. Such research should aim to fill the gaps in available knowledge [66]. In particular, much more data is needed about the interaction of herbal medications with other medications, as well as safety in certain populations, because some patient groups may be more at risk than others [66].

Comparative testing with existing conventional medications can reveal important information about effectiveness, safety, and cost effectiveness. However, for ethical comparative analysis, such comparisons should also include assessment of the broader societal and environmental impacts of sourcing and manufacturing of each comparator, as well as respect for patient preferences.

Herbal medicines make a vast contribution to global healthcare, both as a traditional and a complementary form of treatment, as well as being important sources of new conventional drugs. In spite of this, globally, investment in herbal medicine research is totally out of proportion to global reliance upon plant-based medications. For instance, in the United States, funding for National Center for Complementary and Integrative Health (NCCIH) research activity in 2015 amounted to $124 million while total funding of health research and development activity in the United States amounted to $158,716 million [67], indicating that NCCIH funding is equivalent to less than 0.08% of all medical research funding. Funding for wide scale herbal medicine research, of the type that would be needed to develop a robust evidence base, is simply not available.

However, in some countries, particularly where the use of traditional forms of ethnomedicine are more widespread and culturally embedded, there appears to be a greater inclination and endeavour towards improving quality, regulatory, and ethical standards. For example, in India, the Ministry of AYUSH (Ayurveda, Yoga and Naturopathy, Unani, Siddha and Homeopathy) was established in 2014 to improve standards, inter alia, in education, research, cultivation of medicinal plants, and pharmacopoeia standards of these healthcare systems. The most recent AYUSH annual report (2017-2018) [68] outlines a vision for ‘comprehensive development of conservation, cultivation, collection, processing, marketing, research and extension support system' for medicinal plants. In addition to this aim, a* National Policy on Medicinal Plants* is currently being developed to harmonise the current disparate governance mechanisms under a single code. The same annual report describes India as the world's second largest exporter of medicinal plants after China, with both countries together producing more than 70% of herbal products globally. Hence, it is unsurprising that steps are also being taken in China to improve standards.

In 2016, the State Council of the People's Republic of China published their first white paper on the topic of Traditional Chinese Medicine [69]. Significantly, this paper declares that equal attention will be paid to traditional and conventional (Western) medicine in terms of regulation, training, and practice. The white paper also describes recent efforts to enhance standards in research, in quality and safety, and to promote green and sustainable development, including the enactment of new laws and regulations on the protection of resources in the wild and the protection of rare and endangered species.

Clearly, significant efforts are being made that will improve ethical standards in the use of medicinal plants in certain countries. However, given that the number of medicinal plants is so vast and that the types of plants vary between locations, many challenges remain for ethical herbal medicine globally.

## 6. Conclusion

Application of a simple ethical framework, such as that of the four values, can be used to reveal ethical challenges for herbal medicine but also to suggest strategies for enhancing ethical standards in the production, education, and practice of herbal medicine. We have made suggestions for how the values can be realised within each of these activities, but also key to enhancing ethical practice in herbal medicine is greater investment in research. While certain steps can be taken to ensure that patients are treated with care and respect and that they are informed about the potential consequences of herbal treatment, that information is limited by availability. Given the vast number of plant-based medicines, the acquisition of pharmacological information and the potential for drug interactions pose a major challenge, but failure to address this challenge is tantamount to acceptance of lower ethical standards for those who choose, or those who rely upon, herbal medicines.

Finally, we would like to close with the suggestion that professional bodies, scientists, manufacturers, and ethicists from around the world work closely together to develop a more unified strategy for enhancing ethical standards in herbal medicine globally. The values framework could be used to help frame such a strategy because it can apply equally to all aspects from supply chain through to practice.

## References

[1] Chen S., Pang X., Song J. (2014). A renaissance in herbal medicine identification: From morphology to DNA. *Biotechnology Advances*.

[2] Posadzki P., Watson L. K., Ernst E. (2013). Adverse effects of herbal medicines: An overview of systematic reviews. *Clinical Medicine*.

[3] World Health Organization (WHO) (2013). *WHO Traditional Medicine Strategy: 2014-2023*.

[4] Mills S., Bone K. (2000). Principles and practice of phytotherapy. *Modern Herbal Medicine*.

[5] Quinlan M. B., Singer M., Erickson P. I. (2011). *Ethnomedicine*.

[6] World Health Organization (2008). Traditional Medicine.

[7] Oyebode O., Kandala N.-B., Chilton P. J., Lilford R. J. (2016). Use of traditional medicine in middle-income countries: A WHO-SAGE study. *Health Policy and Planning*.

[8] Hexa Research, Herbal Medicine Market Size and Forecast and Trend Analysis, 2014-2024, https://www.hexaresearch.com/research-report/global-herbal-medicine-market/, 2017

[9] Wheaton A. G., Blanck H. M., Gizlice Z., Reyes M. (2005). Medicinal herb use in a population-based survey of adults: Prevalence and frequency of use, reasons for use, and use among their children. *Annals of Epidemiology*.

[10] Yeh C.-H., Tsai J.-L., Li W. (2000). Use of alternative therapy among pediatric oncology patients in Taiwan. *Pediatric Hematology and Oncology*.

[11] Kennedy J. (2005). Herb and supplement use in the US adult population. *Clinical Therapeutics*.

[12] Abbott R. https://papers.ssrn.com/sol3/papers.cfm?abstract_id=2406649.

[13] Salehi B., Mishra A. P., Shukla I. (2018). Thymol, thyme, and other plant sources: Health and potential uses. *Phytotherapy Research*.

[14] Salehi B., Stojanović-Radić Z., Matejić J. (2018). Plants of genus *Mentha*: from farm to food factory. *Plants*.

[15] Sharifi-Rad M., Nazaruk J., Polito L. (2018). *Matricaria* genus as a source of antimicrobial agents: From farm to pharmacy and food applications. *Microbiological Research*.

[16] Sharifi-Rad J., Tayeboon G. S., Niknam F. (2018). *Veronica persica* Poir. extract – antibacterial, antifungal and scolicidal activities, and inhibitory potential on acetylcholinesterase, tyrosinase, lipoxygenase and xanthine oxidase. *Cellular and Molecular Biology*.

[17] Sharifi-Rad M., Salehi B., Sharifi-Rad J., Setzer W. N., Iriti M. (2018). *Pulicaria vulgaris* Gaertn. essential oil: an alternative or complementary treatment for Leishmaniasis. *Cellular and Molecular Biology*.

[18] Mishra A. P., Saklani S., Sharifi-Rad M. (2018). Antibacterial potential of *Saussurea obvallata* petroleum ether extract: A spiritually revered medicinal plant. *Cellular and Molecular Biology*.

[19] Sharifi-Rad J., Roointan A., Setzer W. N., Sharifi-Rad M., Iriti M., Salehi B. (2018). Susceptibility of *Leishmania major* to *Veronica persica* Poir. extracts - *In vitro* and *in vivo* assays. *Cellular and Molecular Biology*.

[20] Sharifi-Rad J., Sharifi-Rad M., Salehi B. (2018). *In vitro* and *in vivo* assessment of free radical scavenging and antioxidant activities of *Veronica persica* Poir. *Cellular and Molecular Biology*.

[21] Pan Si-Yuan, Zhou Shu-Feng, Gao Si-Hua (2013). New Perspectives on How to Discover Drugs from Herbal Medicines: CAM's Outstanding Contribution to Modern Therapeutics. *Evidence-Based Complementary and Alternative Medicine*.

[22] Harvey A. L. (2008). Natural products in drug discovery. *Drug Discovery Therapy*.

[23] Fabricant D. S., Farnsworth N. R. (2001). The value of plants used in traditional medicine for drug discovery. *Environmental Health Perspectives*.

[24] Jamshidi-Kia F., Lorigooini Z., Amini-Khoei H. (2018). Medicinal plants: Past history and future perspective. *Journal of HerbMed Pharmacology*.

[25] Maiti B., Nagori B., Singh R. (2011). Recent trends in herbal drugs: a review. *International Journal of Drug Research and Technology*.

[26] Khan S. F., Khan R., Qureshi A. W., Zaman S., Khan Z. U. A., Ullah S. (2017). Evaluation of recent trends of prescribing herbal drugs among the prescribers; a pilot study. *International Journal of Basic Medical Sciences and Pharmacy (IJBMSP)*.

[27] Sarris J. (2018). Herbal medicines in the treatment of psychiatric disorders: 10-year updated review. *Phytotherapy Research*.

[28] Salehi B., Sharopov F., Martorell M. (2018). Phytochemicals in *Helicobacter pylori* Infections: What Are We Doing Now?. *International Journal of Molecular Sciences*.

[29] Salehi B., Valussi M., Jugran A. K. (2018). *Nepeta* Species: From Farm to Food applications and Phytotherapy. *Trends in Food Science & Technology*.

[30] Prakash Mishra A., Sharifi-Rad M., Shariati M. A. (2018). Bioactive compounds and health benefits of edible *Rumex* species-A review. *Cellular and Molecular Biology*.

[31] Mishra A. P., Saklani S., Salehi B. (2018). *Satyrium nepalense*, a high altitude medicinal orchid of Indian Himalayan region: chemical profile and biological activities of tuber extracts. *Cellular and Molecular Biology*.

[32] Sharifi-Rad M., Fokou P., Sharopov F. (2018). Antiulcer Agents: From Plant Extracts to Phytochemicals in Healing Promotion. *Molecules*.

[33] Okoronkwo I., Onyia-Pat J.-L., Okpala P., Agbo M.-A., Ndu A. (2014). Patterns of complementary and alternative medicine use, perceived benefits, and adverse effects among adult users in Enugu Urban, Southeast Nigeria. *Evidence-Based Complementary and Alternative Medicine*.

[34] White A., Boon H., Alraek T. (2014). Reducing the risk of complementary and alternative medicine (CAM): Challenges and priorities. *European Journal of Integrative Medicine*.

[35] Werner S. M. (2014). Patient safety and the widespread use of herbs and supplements. *Frontiers in Pharmacology*.

[36] Adams D., Nasser H., Georges S., Vohra S., Gardiner P. (2010). Adverse Events of Medicinal Herbs in Children And Adolescents: A Systematic Review. *Pharmaceutical Biology*.

[37] Gilmour J., Harrison C., Asadi L., Cohen M. H., Vohra S. (2011). Complementary and alternative medicine practitioners' standard of care: Responsibilities to patients and parents. *Pediatrics*.

[38] Smith C. A., Priest R., Carmady B., Bourchier S., Bensoussan A. (2011). The ethics of traditional Chinese and western herbal medicine research: Views of researchers and human ethics committees in Australia. *Evidence-Based Complementary and Alternative Medicine*.

[39] Curtis P., Gaylord S. (2005). Safety Issues in the Interaction of Conventional, Complementary, and Alternative Health Care. *Complementary Health Practice Review*.

[40] Rao M. M., Meena A. K., Galib (2011). Detection of toxic heavy metals and pesticide residue in herbal plants which are commonly used in the herbal formulations. *Environmental Modeling & Assessment*.

[41] Posadzki P., Watson L., Ernst E. (2013). Contamination and adulteration of herbal medicinal products (HMPs): an overview of systematic reviews. *European Journal of Clinical Pharmacology*.

[42] Ernst E., Cohen M. H. (2001). Informed consent in complementary and alternative medicine. *JAMA Internal Medicine*.

[43] Brulotte J., Vohra S. (2008). Epidemiology of NHP-drug interactions: Identification and evaluation. *Current Drug Metabolism*.

[44] Ernst E. (2004). Challenges for phytopharmacovigilance. *Postgraduate Medical Journal*.

[45] Ernst E. (2004). Informed consent: A potential dilemma for complementary medicine. *Journal of Manipulative and Physiological Therapeutics*.

[46] Cohen M. H., Kemper K. J., Stevens L., Hashimoto D., Gilmour J. (2005). Pediatric use of complementary therapies: Ethical and policy choices. *Pediatrics*.

[47] Ernst E. (2009). Advice offered by practitioners of complementary/ alternative medicine: An important ethical issue. *Evaluation & the Health Professions*.

[48] Smith K. R. (2008). Anomalous therapies and public health: A utilitarian bioethical response. *Public Health Nursing*.

[49] Bodeker G., Van'T Klooster C., Weisbord E. (2014). *Prunus africana* (Hook.f.) Kalkman: the overexploitation of a medicinal plant species and its legal context. *The Journal of Alternative and Complementary Medicine*.

[50] Rastogi S., Kaphle K. (2011). Sustainable traditional medicine: taking the inspirations from ancient veterinary science. *Evidence-Based Complementary and Alternative Medicine*.

[51] Ncube B., Finnie J. F., Van Staden J. (2012). Quality from the field: the impact of environmental factors as quality determinants in medicinal plants. *South African Journal of Botany*.

[52] van Andel T., Havinga R. (2008). Sustainability aspects of commercial medicinal plant harvesting in Suriname. *Forest Ecology and Management*.

[53] Bonde S., Firenze P. https://www.brown.edu/academics/science-and-technology-studies/framework-making-ethical-decisions.

[54] Childress J. F., Beauchamp T. L. (2001). *Principles of biomedical ethics*.

[55] Holm S. (1995). Not just autonomy - The principles of American biomedical ethics. *Journal of Medical Ethics*.

[56] Osuji P. I. (2014). *African Traditional Medicine: Autonomy and Informed Consent*.

[57] TRUST Consortium, Global code of conduct for research in resource-poor settings, 2018; http://www.globalcodeofconduct.org/

[58] MacIntyre A. (1984). *After Virtue: A Study in Moral Theory*.

[59] (2017). *San code of research ethics*.

[60] Stuttaford M., Al Makhamreh S., Coomans F., Harrington J., Himonga C., Hundt G. L. (2014). The right to traditional, complementary, and alternative health care. *Global Health Action*.

[61] Njuguna F., Mostert S., Seijffert A. (2015). Parental experiences of childhood cancer treatment in Kenya. *Supportive Care in Cancer*.

[62] Sato A. (2012). Do Inequalities in Health Care Utilization in Developing Countries Change When We Take into Account Traditional Medicines?. *World Development*.

[63] Uppsala Monitoring Centre What is VigiBase?. https://www.who-umc.org/vigibase/vigibase/.

[64] Sugarman J., Burk L. (1998). Physicians' ethical obligations regarding alternative medicine. *Journal of the American Medical Association*.

[65] Adams K. E., Cohen M. H., Eisenberg D., Jonsen A. R. (2002). Ethical considerations of complementary and alternative medical therapies in conventional medical settings. *Annals of Internal Medicine*.

[66] Matthews H. B., Lucier G. W., Fisher K. D. (1999). Medicinal herbs in the United States: Research needs. *Environmental Health Perspectives*.

[67] Research America (2016). *US investments in medical and health research and development*.

[68] AYUSH (2018). *Annual Report 2017-2018*.

[69] The State Council of the People's Republic of China (2016). *Traditional Chinese Medicine in China*.

